# Autonomous Road Roundabout Detection and Navigation System for Smart Vehicles and Cities Using Laser Simulator–Fuzzy Logic Algorithms and Sensor Fusion

**DOI:** 10.3390/s20133694

**Published:** 2020-07-01

**Authors:** Mohammed A. H. Ali, Musa Mailah, Waheb A. Jabbar, Khaja Moiduddin, Wadea Ameen, Hisham Alkhalefah

**Affiliations:** 1 Faculty of Manufacturing Engineering, Universiti Malaysia Pahang (UMP), Pekan 26600, Malaysia; 2 School of Mechanical Engineering, Faculty of Engineering, Universiti Teknologi Malaysia, UTM Johor Bahru 81310, Malaysia; musa@fkm.utm.my; 3 Faculty of Electrical and Electronic Engineering Technology, Universiti Malaysia Pahang (UMP), Pekan 26600, Malaysia; waheb@ump.edu.my; 4 Advanced Manufacturing Institute, King Saud University, Riyadh 11451, Saudi Arabia; khussain1@ksu.edu.sa (K.M.); wqaid@ksu.edu.sa (W.A.); halkhalefah@ksu.edu.sa (H.A.)

**Keywords:** wheeled mobile robot, path panning, laser simulator, fuzzy logic, laser range finder, Wi-Fi camera, sensor fusion, local map, odometry

## Abstract

A real-time roundabout detection and navigation system for smart vehicles and cities using laser simulator–fuzzy logic algorithms and sensor fusion in a road environment is presented in this paper. A wheeled mobile robot (WMR) is supposed to navigate autonomously on the road in real-time and reach a predefined goal while discovering and detecting the road roundabout. A complete modeling and path planning of the road’s roundabout intersection was derived to enable the WMR to navigate autonomously in indoor and outdoor terrains. A new algorithm, called Laser Simulator, has been introduced to detect various entities in a road roundabout setting, which is later integrated with fuzzy logic algorithm for making the right decision about the existence of the roundabout. The sensor fusion process involving the use of a Wi-Fi camera, laser range finder, and odometry was implemented to generate the robot’s path planning and localization within the road environment. The local maps were built using the extracted data from the camera and laser range finder to estimate the road parameters such as road width, side curbs, and roundabout center, all in two-dimensional space. The path generation algorithm was fully derived within the local maps and tested with a WMR platform in real-time.

## 1. Introduction

The navigation of autonomous vehicle in urban-building and roads is still a tricky topic in robotics research worldwide, due to many uncertain cases that prevail while navigating on roads. The autonomous vehicle is currently not just used as a transportation medium but can be also utilized for performing some road services like road marks painting, grass cutting, and side-road cleaning [1,2,3].

A complete navigation algorithm that can consider and deal with all the conditions encountered in the road environment has yet to be thoroughly constructed and developed [4]. One such case is navigation and path determination in the open space area, e.g., in a roundabout and T, Y, and cross junctions. Three main challenges are likely to be encountered during navigation in such areas: firstly, the capability to detect the printed marks during weather changes; secondly is the ability to discover and analyze the traffic light signals; and finally, to detect various intersections or junctions using the natural landmarks like borders and edges in such areas, when the printed road marks are missed.

The open space road areas include branched/unbranched roads [5,6] and road junctions/intersections [7,8,9,10,11,12,13]. It has been remarked that a limited number of studies have addressed the autonomous navigation of mobile robot in open-space areas of the roads such as cross, roundabout, T, and Y intersections. Another situation for open space area on the roads can be seen when there is a sudden change of the road width along the span of roads [13].

Several works for navigation on the road intersections (e.g., T and Y branches and cross areas) and road following, have been reported [7,8,9,10,11,12,13,14,15,16,17,18] through building a suitable local map of terrains using sensors fusion technique. In fact, a handful of the above-mentioned reported works have dealt with a roundabout environment where the surroundings and rules of the road are significantly different from other kinds of junctions or intersections. The roundabout intersection typically consists of a circular area in the middle and several inlet/outlet branches on the sides, which enables the vehicles to rotate around until finding the suitable exit branch without a need for traffic light signals, such as in the cross junction, where the vehicle selects accordingly its exit branch based on appropriate traffic light signals.

## 2. Related Works

The open space areas such as T, Y, cross, and roundabouts intersections are considered as the main challenge during autonomous vehicle navigation on the roads, due to sudden changes of the road’s direction, losses of sensors’ signals, and difficulty to make the right decision in such environments. Other open space area challenges can be found during autonomous navigation and right maneuver searching in underwater vehicles [19,20,21].

This paper focuses on the road roundabout environments navigation, since it needs a further development and consideration as stated in Section 1; however, the other types of junctions such as T/Y and cross intersections have been already studied well in the literature [7,8,9,10,11,12,13,14,15,16,17,18].

Most of the current methods used for roundabout navigation and detection depend on the offline information coming from GPS and maps to recognize the roundabout and find the proper exit of roundabout. Jorge et al. [22] have developed an algorithm to recognize the roundabout setting and find the proper maneuvers using so-called open street map and GPS, which help to find the appropriate direction of vehicle to be undertaken by the driver either in left, right, and straight manners. In fact, this is a manual and offline navigation system that utilizes a yaw-rate profile to determine the exit of roundabout. A 3D simulation of CyberCar to detect a roundabout setting from well-known digital maps is developed by Rastelli et al. [23]. In this simulator, once the car arrives at the entrance of the roundabout setting, it generates a circular path in the map with a radius slightly larger than the roundabout radius.

A steering system to maintain the vehicle on a circular path during navigation in roundabout using a fusion of fuzzy-logic controller with distance curve gain and angular-error strategies has been proposed by Katrakazas et al. [24] and Rastelli et al. [25]. In addition, a control system using inertial measurement unit (IMU) and GPS is used for roundabout trajectory tracking. In fact, the features of a roundabout are completely known, such as center, entrance, and exit of the roundabout from GPS and the maps data; however, the path generation is determined from inlet to outlet of roundabout using Bezier curves strategy.

A guidance system that allows the vehicle to travel along an optimum traffic lane in a roundabout setting and passes the information to the driver has been developed by Okusa et al. [26]. In this system, the maps and GPS are used to localize the car in the roundabout setting; however, the traffic signals and vehicle turning in roundabouts are determined using a group of sensor data that can be generally classified into two subgroups, namely; traveling distance and direction sensors.

Laura et al. [27] has presented a machine learning algorithm-based predictive model to estimate the vehicle speed and steering angles in a roundabout intersection. In fact, this model depends on Open-Streets-Maps and recorded video to identify the roundabout geometry and dimensions, which is unsafe for driving in such a dangerous area and is subject to errors; thus there is a need for online estimation of roundabout parameters.

A sensor fusion model has been used to predict 3D dimensions of surrounding environments during autonomous driving on roundabouts using LIDAR point’s cloud and camera [28]. It builds a 3D objects map by integrating the data of the images with LIDAR using 3D bounding boxes. This work is just focused on the roundabout environment detection; however, it does not show how to generate the path within the roundabout environment. A 2D image has been combined with 3D point cloud to recognize the road’s traffic signs for the purpose of autonomous transportation systems [29]. It uses the bag of visual phrases and Gaussian Bernoulli deep Boltzmann algorithm to extract the features of the traffic signs of the road environment, which is useful for detection of traffic signs; however, it is not able to generate the path within the road environments.

Tesla and Google autonomous cars have used a sensor fusion technique for building a 3D map of a vehicle’s surrounding environments and finding the cars located at the back or side of the autonomous vehicle [30]. It uses velodyne laser range finder (LRF) for construction of an online map, camera for estimating the obstacles, car on the side or back of the autonomous car, and GPS with Google maps to localize the vehicle within the road environments from the start to goal positions. Such autonomous vehicles depend mainly on GPS and Google maps to recognize the roundabout geometry, which might cause damage to pedestrians and road infrastructure when there is a recent change on the roundabout that has not been yet registered on the Google maps or there are signal losses in GPS sensor.

Owing to the accuracy and safety issues such as incapability to identify a roundabout in a specific location using GPS, GPS signal losses, and issues in recognizing the unexpected changes of the road roundabout terrain, it may result in particular damage and unwarranted harm to the road infrastructure and pedestrians. Hence, there is a necessity to utilize onboard sensors such as camera, odometry, and LRF for online detection, path determination, and decision making during navigation of roundabout environments. It is deemed to be amongst the pioneering researches that advocates the implantation of real-time navigation in a road roundabout environment using cameras, odometry, and LRF sensors. This paper presents an onboard navigation system in a roundabout using a novel algorithm, called Laser Simulator (LS), which is integrated with a fuzzy logic (FL) algorithm and sensor fusion for autonomous navigation in roundabout environments. The combination between LS and FL algorithms was introduced to make the right decision on the existence of the roundabout, with the sensor fusion implemented to determine the appropriate robot path in a roundabout environment.

In this work, the robot is navigating autonomously in the road following and later moves effectively in a circular path of a standard roundabout. It finds the path starting from the predefined initial position until it reaches the predefined goal using the LS–FL algorithms and sensor fusion. This approach was implemented for recognizing the roundabout within the local map with sensor fusion involving the LRF and odometry measurements to determine the robot path. The predefined initial and goal positions were determined using a DGPS system and the robot uses such information to detect the direction of the roundabout exit. Because the robot is navigating in a relatively small area (within 10–500 m diameter), it is not useful to use GPS, and instead, the robot could be informed about the roundabout exit direction and goal position even prior to its activation. The goal position can be determined in terms of the distance the robot should travel after passing the roundabout, e.g., robot to proceed to the east direction of the roundabout (270°) for about 20 m distance to a goal position.

In this work, two tasks were performed to enable the mobile robot to pass autonomously through the roundabout:

1. Autonomous road roundabout detection
2. Autonomous road roundabout navigation

The details of each task are described in the subsequent sections.

## 3. Autonomous Road Roundabout Detection

It is not an easy task to decide if the circular/elliptical object located ahead of the robot during navigation is indeed a road roundabout or otherwise. Thus, a decision making procedure is needed to distinguish clearly the roundabout and other similar circular/elliptical shaped objects, such as obstacles, vehicles, etc. It is worthwhile to note that the Google maps and GPS are also currently and typically used in conjunction with many navigation systems to identify a roundabout location [23]. However, these methods have some major limitations on the accuracy and safety issues previously mentioned that may contribute to adverse effects and undesired consequences to road users, pedestrians and road curbs. Thus, the proposed study is regarded as an attempt to implement an online detection and navigation system in a roundabout setting using LRF, camera, and odometry sensors.

**Figure 1:**
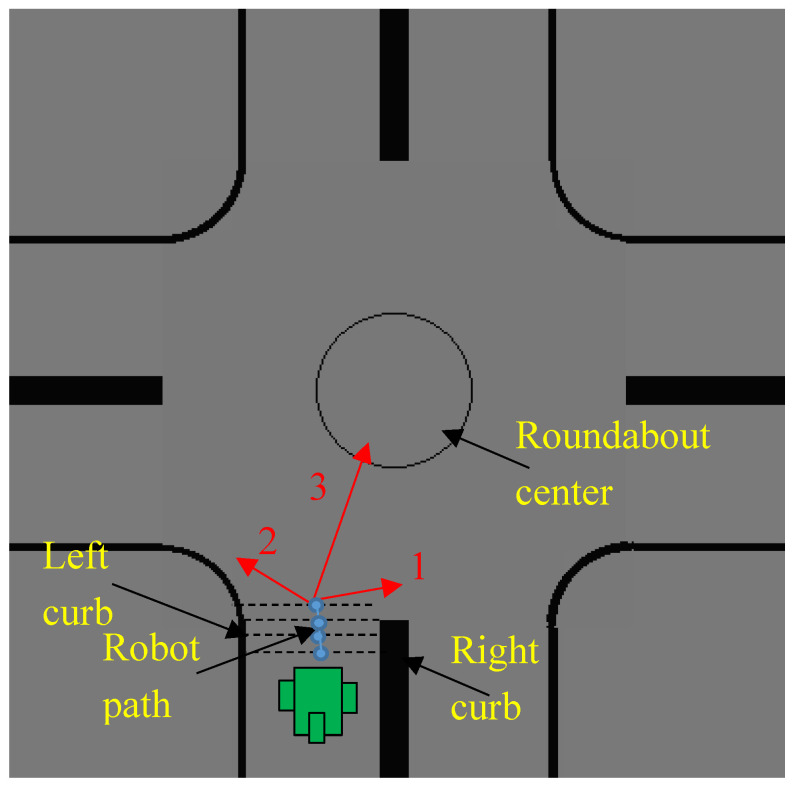
Conditions of the roundabout used for roundabout detection: (1) right curb is faded, (2) left curb is slightly faded, and (3) circular path is detected.

Three roundabout conditions were considered to detect the presence of a roundabout ahead of the robot during autonomous navigation on the road, as shown in Figure 1, assuming a right hand drive convention:

1. The right curb of the road is suddenly faded
2. The left curb of the road is slightly faded
3. There is a circular/elliptical curve located in front of the robot

All these conditions should occur simultaneously.

Prior to encountering the above conditions, a local map has to be created for the road environment using a camera and an image processing algorithm as follows:

### 3.1. Developing of a Local Map for the Road Environment

**Figure 2:**
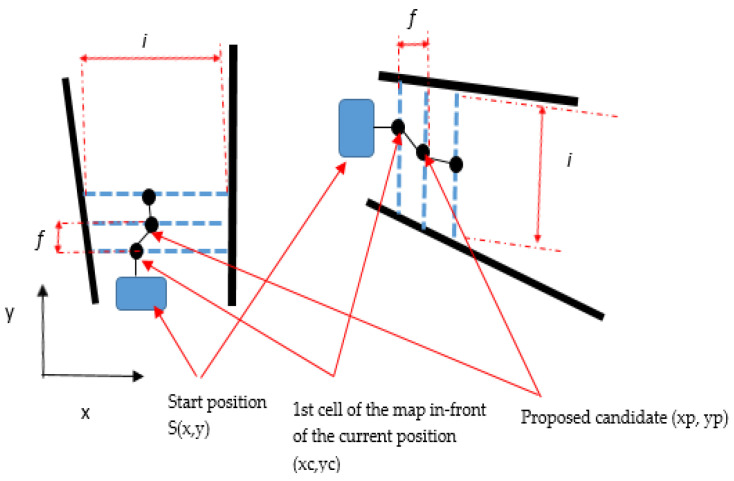
Basics of the Laser Simulator (LS) principle.

This was first done by performing video streaming. It was captured by a camera with suitable resolution, and then the captured image was processed using MATLAB software involving suitable image acquisition and processing toolboxes to perform an online image capturing and processing procedure. The image processing algorithm has been used to extract the road environment features and build the local map using laser simulator algorithm as shown in Figure 2.

The brightness of the image sequences was adjusted, since the setting of the camera aperture is not automatic. The following operations were applied selectively for edge detection and noise filtering: 2D Gaussian filters (fspecial), multidimensional images (imfilter), canny edge algorithm, morphological structuring element (strel), dilating image (imdilate), 2D order-statistic filtering (ordfil2), removing small objects from binary image (bwareaopen), and filling image regions and holes (imfill). The results of the image processing algorithm and local mapping of the road environment will be shown in Section 3.2.

After building the local map of the road, the subsequent image post processing procedure was implemented to check whether the object located in front of the robot is a roundabout or otherwise as follows.

### 3.2. Laser Simulator-Based Roundabout Detection

A new algorithm for observing the road curbs and roundabout in the developed local maps using the so-called LS algorithm integrated with FL algorithm has been introduced in this section. It is actually emulating the laser beams by sending a series of points as horizontal or vertical lines in the local map that has been prepared in the previous step as shown in Figure 2, to detect either the faded road curbs or the location of a roundabout center. This will be followed by the application of fuzzy logic algorithm to make the right decision in regard to roundabout detection.

#### 3.2.1. Right and Left Side Detection

The subsequent algorithm has been used to detect the road curbs in the camera’s local map:

##### Generation of Points’ Center Reference in the Image

**Figure 3:**
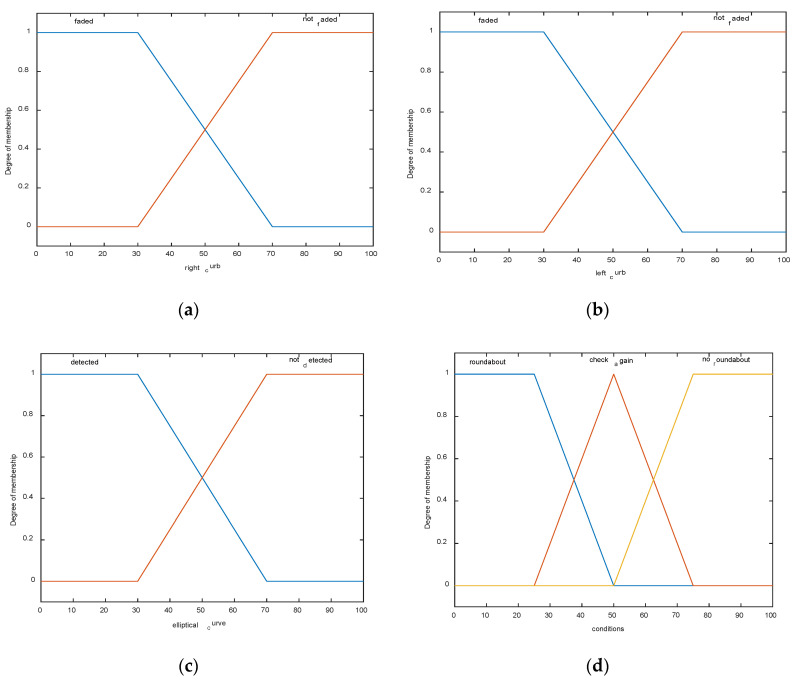
Input/output fuzzy membership functions: (**a**) input: right curb, (**b**) input: left curb, (**c**) input: elliptical curve, and (**d**) output: conditions.

**Figure 4:**
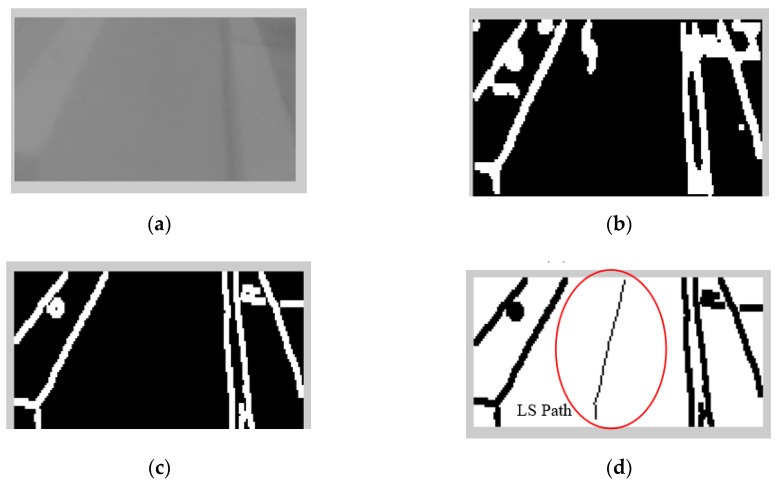
Image sequences processing where no roundabout can be detected using LS: (**a**) original image gray scale, (**b**) image after applying curbs detection, (**c**) image after removing the noise, and (**d**) image after applying the LS (continuous line in the middle).

**Figure 5:**
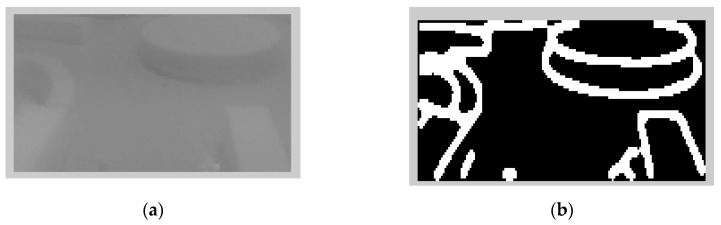

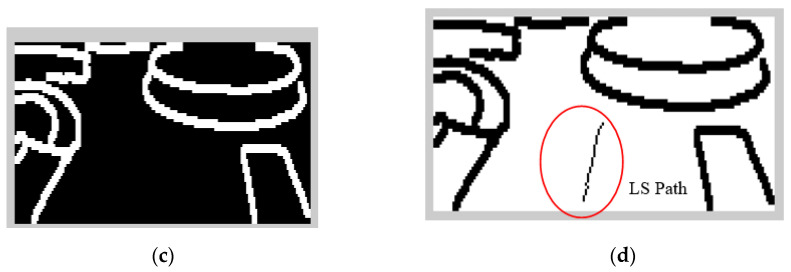
Image sequences processing where roundabout is detected using Laser Simulator: (**a**) original image in gray scale, (**b**) image after applying curbs and roundabout detection, (**c**) image after removing the noise, and (**d**) image after applying the LS (discontinuous line in the middle).

Because the robot with its camera was assumed to be placed between the road sides as depicted in Figure 3, Figure 4 and Figure 5, the position of the camera has been located in the middle of the image’s frame sequence, exactly in bottom horizontal area of each image’s frame. This is the 1st point’s reference center c(x,y). It is followed by next points’ reference centers which are produced by series points as horizontal line as in Equation (1):

(1) $$y=x_{c}+i$$

where *i* is an incremental value located between 1 and the image’s height (image’s resolution in pixels in *y*-direction). The horizontal lines lengths are chosen between the subsequent limits as expressed in Equation (2):

*y_right_* = *y_c_* + *R_p_*; *y_left_* = *y_c_* − *L_p_* (2)

where *y_right_* is the limit of the lines on the right side. *y_left_* is the lines’ limit on the left-side. *R_p_* is the pixels’ number starting from the center to the curb of right side. *L_p_* is pixels’ number starting from the center to the curb of left side. The reference center points are then described as in Equation (3):

*x_cnew_* = *x_c_* − *i*; *y_cnew_* = *y_left_* + (*R_p_* + *L_p_*)/2 (3)

##### Detecting of the Curbs in the Right and Left Sides

To detect the road curbs, a series of points as inclined lines with slight changes of angles were produced in the image’s frame in the video, initiating from the points centers *x_cnew_* and *y_cnew_* that have been calculated in Equation (3) and shown in Figure 2. The equations that are used to determine these lines can be written as follows:

With the left curb of the points’ centers *x_cnew_* and *y_cnew_*, these lines were generated by:

*x_L_* = *x_cnew_* − *f*; *y_L_* = *y_cnew_* − (*x_cnew_* − *x_L_*) tan(*δ*) (4)

And they are generated for the right curb of the points’ centers *x_cnew_* and *y_cnew_* by:

*x_r_* = *x_cnew_* + *k_r_*; *y_r_* = *y_cnew_* − (*x_r_* − *x_cnew_*) tan(*δ*) (5)

where *f* = 1:*L*_p_. *k_r_* = 1:*R*_p_. *L_p_* and *R_p_* are the pixels’ number which are existed between the points’ centers *x_cnew_* and *y_cnew_* and right or left side, in respectively. *δ* is the slope of inclined lines to the ground. The number of pixels (*P*) for Equation (4) can be expressed as:

(6) $$P_{L}=\sum_{j=1}^{L_{P}}P_{j}$$

The number of pixels (*P*) for Equation (5) can be expressed as:

(7) $$P_{r}=\sum_{k=1}^{L_{P}}P_{k}$$

Because the dimension between the road sides as in actual case is almost equal; the pixels in each two successive inclined lines are compared with each other as follows:

(8) $$Al=pl_{i}-pl_{i-1}\;Ar=pl_{i}-pl_{i-1}$$

If *Al* and *Ar* override the threshold as calculated in Equation (9), it indicates that such new line is not belonged to the road side or curb:

(9) $$Al<A_{l1}+d\;Ar<A_{r1}+d$$

where *A_r_*_1_ and *A_l_*_1_ are the 1st tangent line’s pixels in Equations (4) and (5). *d* is a value measure as a ratio of image resolution. Here in this work, this ratio was considered as 10% in both *x* and *y* directions.

#### 3.2.2. Roundabout Center Detection:

The road roundabout presents in a camera’s local map as an ellipse. If the curbs of road could not be determined in the former step, the roundabout detection algorithm will start to be executed. From the reference center point, the algorithm generates multi-tangent lines on the right curb with an angle ranging between 0 until 90 till they intersect with edges. The intersection points between these tangent lines and roundabout center that is represented as an ellipse were calculated. If the intersected points can verify the equation of ellipse as illustrated in Equation (10), it indicates that the center of roundabout center has been detected.

(10) $$\frac{{\left(x-x_{0}\right)}^{2}}{a^{2}}+\frac{{\left(y-y_{0}\right)}^{2}}{b^{2}}=1$$

One can choose four points from intersection points, the first two points could be used to calculate the constants *a* and *b* in Equation (11) and the rest two points for the verification if the shape is an ellipse, which must be achieved based on the following conditions:

(11) $$comp=\frac{x^{2}}{a^{2}}+\frac{y^{2}}{b^{2}{}_{}}-1<ad$$

where *comp* = 0, *ad* represents the allowed deviation from zero (in the program, *ad* = *10*).

### 3.3. Fuzzy Logic-Based Decision Making

Once the three features of the roundabout are determined using the LS (faded right and left curbs and roundabout center detection), the fuzzy logic algorithm is applied subsequently to make the right decision on the roundabout detection. Three fuzzy sets are used as input sets, namely; right_curb, left_curb, and elliptical curve with one as output set called conditions. The input sets have been fuzzified into the linguistic variables as follows: right-curb = {faded (less or equal to *d* as in Equation (9), not_faded (bigger than *d* as in Equation (9))}, left-curb = {not faded (less or equal to *d* as in Equation (9), faded (bigger than *d* as in Equation (9))}, and elliptical curve = {not detected (*comp* > *ad* as in Equation (11)), detected (*comp* < *ad* as in Equation (11))}. The output fuzzy set has been fuzzified into the following linguistic variables: conditions = {roundabout (all condition have been verified), check again (only one condition hasn’t been verified, no roundabout (two conditions out of three haven’t been verified)}. The membership functions of the input and output sets are illustrated in Figure 3.

Four rules have been used in fuzzy logic to identify the existence of the roundabout as follows:

IF (the right curb is faded) and (elliptical curve is detected) and (left curb is faded) THEN (there is a roundabout in front.)
IF (the right curb is faded) and (elliptical curve is detected) and (left curb is not faded) THEN (check again in the next laser simulator lines)
IF (the right curb is not faded) and (elliptical curve is detected) and (left curb is faded) THEN (check again in the next laser simulator lines).
IF (the right curb is not faded) and (elliptical curve is not detected) and (left curb is faded) THEN (this is not a roundabout).

**Figure 6:**
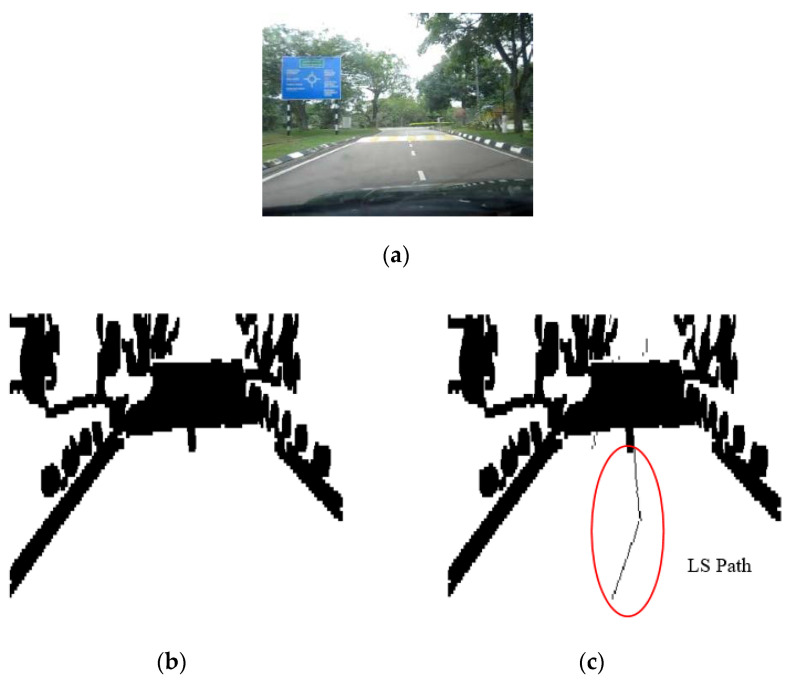
Image sequence with applying LS for roundabout determination at 100 m from the roundabout: (**a**) original image, (**b**) processing image, and (**c**) implementation of LS (continuous dotted line at the middle).

**Figure 7:**
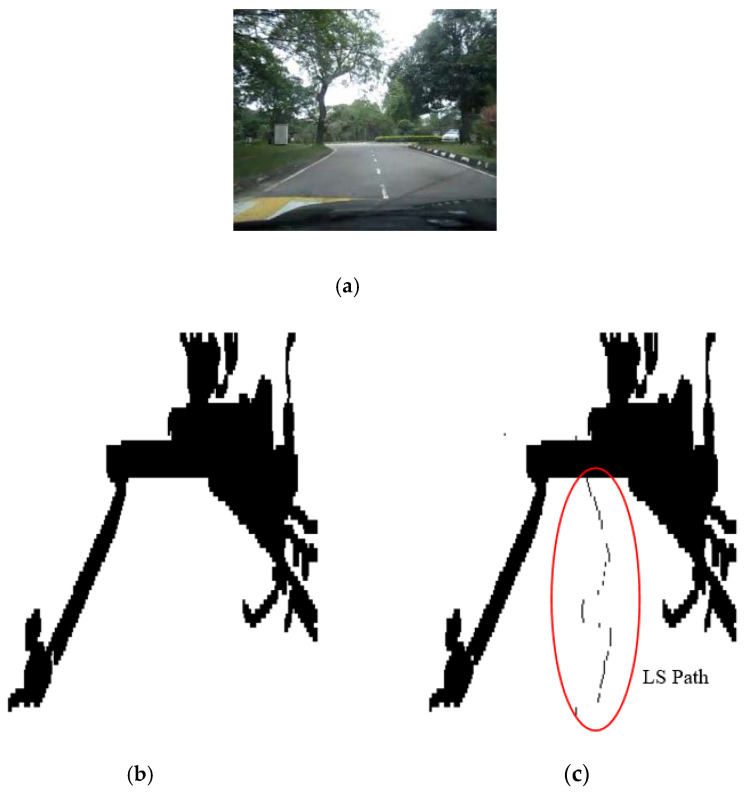
Image sequence with applying LS for roundabout determination at 50 m from the roundabout: (**a**) original image, (**b**) processing image, and (**c**) implementation of LS (continuous dotted line at the middle).

**Figure 8:**
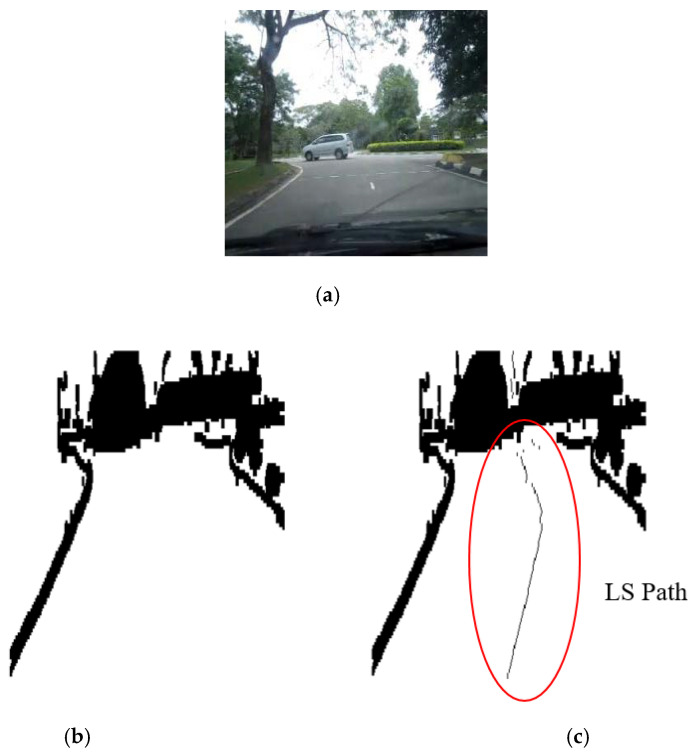
Image sequence with applying LS for roundabout determination at 10 m from the roundabout: (**a**) original image, (**b**) processing image, and (**c**) implementation of LS (continuous dotted line at the middle).

**Figure 9:**
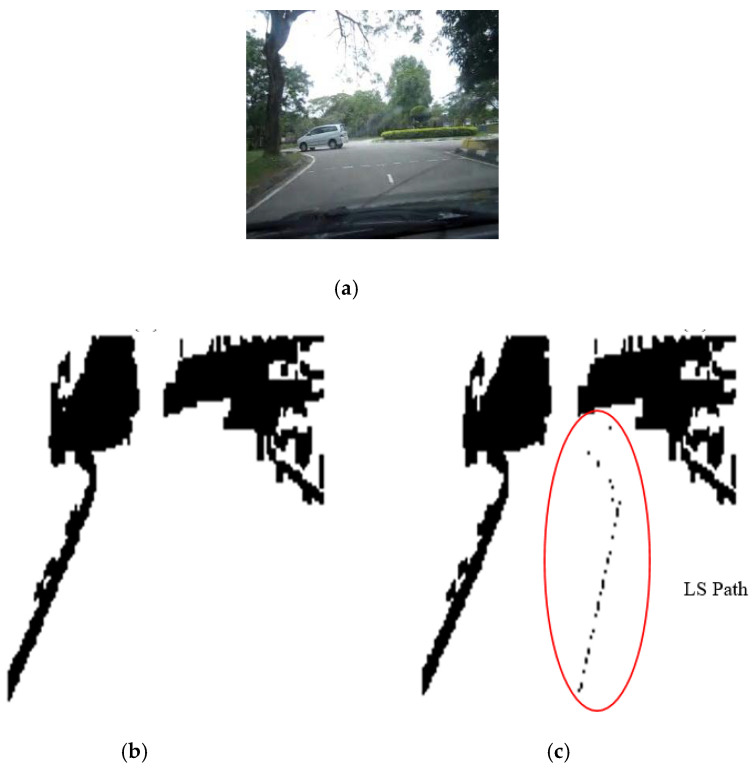
Image sequence with applying LS for roundabout determination at close distance to the roundabout: (**a**) original image, (**b**) processing image, and (**c**) implementation of (continuous dotted line at the middle).

The results of the LS with rules decision are shown in Figure 4d, Figure 5d, Figure 6, Figure 7, Figure 8 and Figure 9c. The continuous lines in the middle of the LS images as shown in Figure 4d denote that the roundabout has not occurred yet; however, the discontinuous line in Figure 5d indicates the detection of roundabout.

The LS algorithm for the roundabout detection has been applied to the real roundabout as shown in Figure 6, Figure 7, Figure 8 and Figure 9. It was observed that the algorithm can effectively detect the existence of the roundabout from the sequences of the images as shown in Figure 6, Figure 7, Figure 8 and Figure 9.

As previously mentioned, the continuous lines in the middle of the image after applying the LS algorithm as shown in the results of Figure 6, Figure 7 and Figure 8c denote that the roundabout has yet to exist; however, the discontinuous line in Figure 9c indicates the early detection of the roundabout.

## 4. Sensor Fusion for Path Planning and Roundabout Navigation

**Figure 10:**
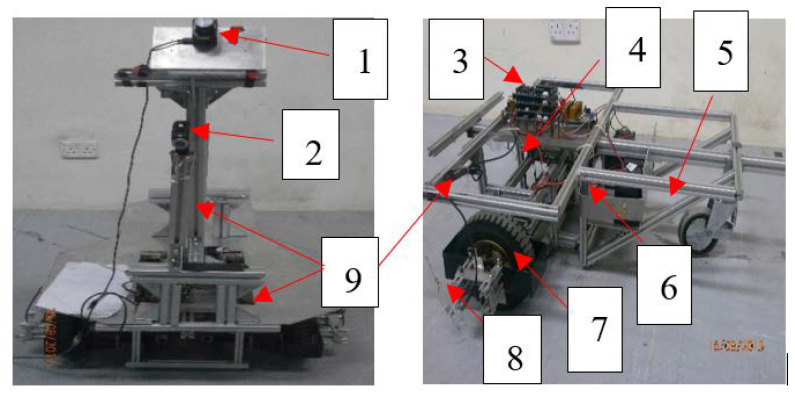
Developed wheeled mobile robot (WMR) platform in this research: (1) LRF, (2) Wi-Fi camera, (3) interface free controller cards, (4) DC-motors driver card, (5) castor wheel, (6) battery, (7) differential drive wheels, (8) rotary encoder, and (9) aluminum profiles and plates.

A wheeled mobile robot (WMR) platform, equipped with LRF, camera, and odometry sensors, has been developed in the laboratory as shown in Figure 10 to accomplish the autonomous navigation in the roundabout intersection.

A sensor fusion technique involving the use of LRF, camera, and odometry has been used to determine the robot path starting from a specific entrance to an appropriate exit of the roundabout. As mentioned in Section 2–A–1, the camera’s image processing algorithm has been used to extract some features of the road curbs and roundabout, e.g., the road roundabout center and borders. Other features will be extracted using the LRF and odometry sensors.

### 4.1. Sensor Fusion Modeling

In this section, the road features extracted by the LRF and odometry sensor will be explained in detail, as follows:

#### 4.1.1. Odometry-Based Measurements

Two encoders were utilized to determine the robot position. They have been linked to the two differential wheels through the encoder’s pins in dual brush card motor. The complete rotation of this rotary encoder is around 500 pulses/rotation; thus the linear displacement can be computed as follows:

(12) $$C=\frac{2\pi \;rP_{cur}}{P_{fr}}$$

where *P_fr_* and *P_cur_* are the pulses of the complete and current rotation, respectively.

#### 4.1.2. LRF-Based Measurements

**Figure 11:**
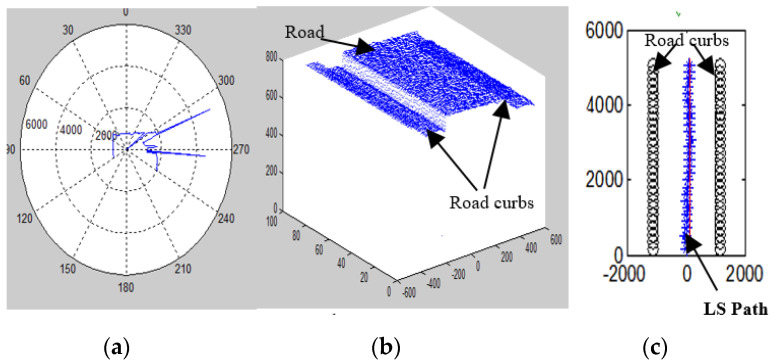
Principle of LRF measurement and calculation: (**a**) one scan measurement (mm), (**b**) road with curbs in 3D (mm), and (**c**) LS path generation (mm).

The LRF measurements were used for building a 2D local map as shown in Figure 11.

Two parameters can be extracted from the LRF measurements as follows:

The road fluctuations (height of objects with respect to the laser device) can be expressed as:

*rf_n_* = *r_n_* cos*θ* (13)

The road width (side distance measurement with respect to the laser device) is presented as:

*rw_n_* = *r_n_* sin*θ* (14)

where *r_n_* is the length of the LRF signal for *n*-th LRF measurement, *θ* (−120°, 0, 120°) is the angle of the laser beam deviation from extreme right at 120° to extreme left at −120°.

If *rf*_0_ is the road fluctuation at *θ* = 0°, which is used as a reference point, *rf_i_* is the other fluctuation measurements located on the left and right side of this point are being compared, and *d* is the threshold of the curb detection, which was set to 10 cm in this work, then:

*rf_i_* − *rf*_0_ ≥ ± *d* (15)

This implies that if the deviation between the reference point and other measured values exceeds the predefined threshold value, then this point is considered as the road curb or obstacles as in Figure 11b. This operation is repeated with all measurements as in Equation (15).

### 4.2. Road Roundabout Navigation

The sensor fusion data were used to enable the robot to navigate autonomously in the roundabout setting starting from the entrance to the exit sections of the roundabout. The algorithm used for driving the robot on the path approaching the entrance or after the exit areas of the roundabout is called road following, whereas the road roundabout center algorithm was used to navigate about the center of the roundabout. This is described as follows:

#### 4.2.1. Navigation in the Road Following

The sensor fusion including camera, LRF and encoders were used effectively to determine the collision-free path in the road following areas. The camera was used to recognize the roundabout area when it exists by LS algorithm as expressed in Section 3.2. The LRF was used for finding the curbs of roads and determine the location of the robot within its environment. The measurement of encoder was utilized to estimate the location of robot during navigation in the given environment.

**Figure 12:**
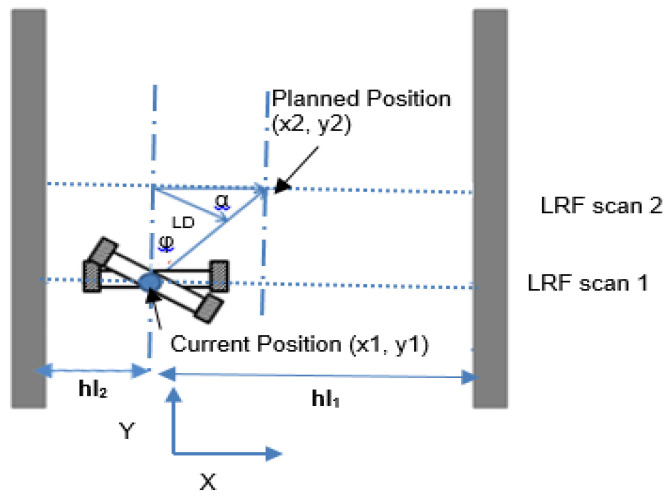
Robot path planning calculation for road following section.

The path determination of the robot using LRF and odometry is discussed as follows:

If the robot’s start position is (*x*_1_, *y*_1_) as depicted in Figure 12, the LS algorithm will determine the planned pose *x*_2_ as a center of the LRF measurements within the two curbs of the road as presented in Equation (15). *y*_2_ is computed from the measurement of encoder as shown in Equation (18).

In Figure 12, *hl*_1_ and *hl*_2_ are the distances between the current position and road curbs in *x*.

The differential wheels of WMR should be moved by an angular velocity as shown in Equation (16):

(16) $$\overset{\cdot }{\varphi }=\frac{V_{r}-V_{l}}{b}\;\;\;\;\;\;\overset{\cdot }{\varphi }b=V_{r}-V_{l}$$

where *V_l_* and *V_r_* are the velocities of the wheels in the left and right sides, respectively, and *b* is the dimension between the right and left wheels in the differential drive mechanism. The left side of Equation (16) can be written as in Equation (17):

(17) $$\overset{\cdot }{\varphi }b=b\frac{\varphi_{\max}-\varphi_{0}}{T}=\frac{b\varphi_{\max}}{T}=\frac{LD}{T}$$

where *LD* is the displacement required to be shifted by WMR to reach the required position starting from the current measurement of LRF as illustrated in Figure 12. *T* is the periodic time of the acuring the measurement, *φ*_0_ is current position heading angle (set at the beginning as 0), and *φ*_max_ is the heading angle’s new location.

The planned position (*x*_2_, *y*_2_) can be calculated as follows:

(18) $$x_{2}=x_{1}+|hl_{2}+hl_{1}|Ra\;y_{2}=y_{1}+\Delta T.\;V\;\mathrm{where}\;V=\frac{V_{r}+V_{l}}{2}$$

where *x_1_* and *y_1_* indicate the current position of the robot, and *Ra* indicates the place where WMR is planning to drive. If *Ra* is setup to 0.5, that means the robot will drive in the mid-distance between the road curbs.

*φ*_max_ in Equation (17) can be further calculated as:

(19) $$\overset{}{\varphi }=\tan^{-1}(\frac{x_{2}}{y_{2}})$$

The rotational distance *LD* can then be calculated as follows:

(20) $$LD=\;(x_{2}-x_{1})\frac{\sin(\alpha )}{\sin(\frac{\pi +\varphi }{2})}\;\mathrm{where}\;\alpha =\frac{\pi }{2}-\varphi$$

#### 4.2.2. Navigation in Road Roundabout Center

When the LS integrated with FL algorithm indicates that the roundabout is located in the camera’s local map, the camera will be shut-off and the algorithm for approaching the roundabout entrance starts to operate. The algorithm for the roundabout entrance is operating based on a mixed information coming from the last camera local map calculation and the last measurements from the LRF.

**Figure 13:**
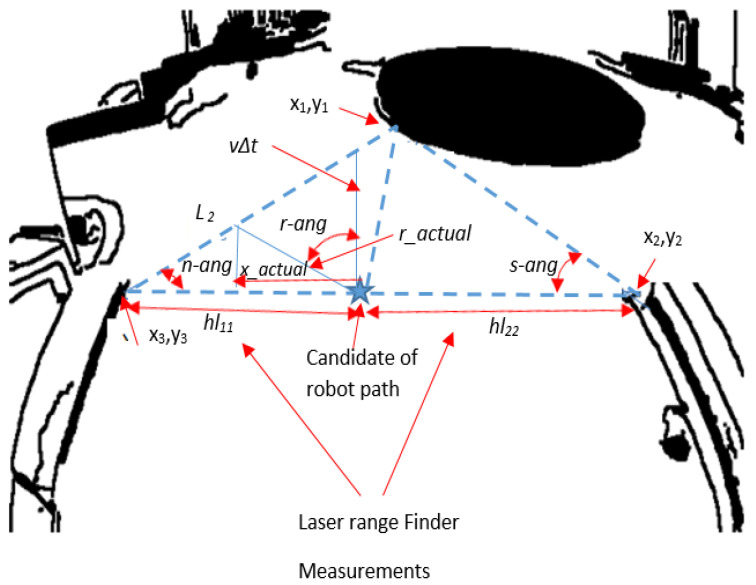
Entrance parameters and path determination of roundabout.

In Figure 13, *s-ang* is the slip angle of the robot, *r–ang* is the rotation angle, and *n-ang* is navigation angle. All these angles can be calculated using Equation (21) from the camera local map, since the coordinates of (x_1_, y_1_), (x_2_, y_2_), and (x_3_, y_3_) are known from the LS algorithm.

(21) $$s\_ang=\arctan\frac{x_{2}-x_{1}}{y_{1}-y_{2}}\;n\_ang=\arctan\frac{x_{3}-x_{1}}{y_{1}-y_{3}}\;r\_ang=\frac{\pi }{2}-s\_ang$$

The robot position, *x* can be computed based on the angles that have been calculated from the camera local map and the last LRF measurements in the road following:

(22) $$x\_actual\;\tan(s\_ang)=(hl_{11}-x\_actual)\tan(n\_ang)$$

where *hl*_11_ is the left measurements of the robot in the road following part as defined in Figure 13.

The *x* and *y* of the planned position for the robot can be then calculated as follows:

(23) $$x\_actual=hl_{11}\frac{\tan(n\_ang)}{\tan(s\_ang)+\tan(n\_ang)}\;Y_{}=\Delta T.\;V\;\mathrm{where}\;V=\frac{V_{r}+V_{L}}{2}$$

The angles of the roundabout entrance were calculated from the camera local map as shown in Figure 13; however, the dimensions were calculated from the last measurements of the LRF. The entrance parameters are illustrated in Figure 13.

The robot angular velocities to reach the planned path can be calculated as:

(24) $$V_{l}-V_{r}=\overset{\cdot }{\varphi }b=b\frac{\varphi_{\max}-\varphi_{0}}{t_{\max}-t_{0}}=\frac{b\varphi_{\max}}{T}=\frac{V\;\sin(r\_ang)}{\sin\;(\;s\_ang+n\_ang)}$$

The planned path of the mobile robot must be in the direction of *r_actual*, which can be calculated as:

(25) $$r\_actual=\frac{x\_actual}{\cos(s\_ang)}$$

If the robot can move by an angle *r*_*ang* and reach the *r_actual*, this means that the robot has reached and passed the roundabout entrance.

The calculation of the exit parameters is almost similar to that of the entrance counterpart. It is in fact exactly an inverse calculation of the entrance if the roundabout is assumed to be standard.

**Figure 14:**
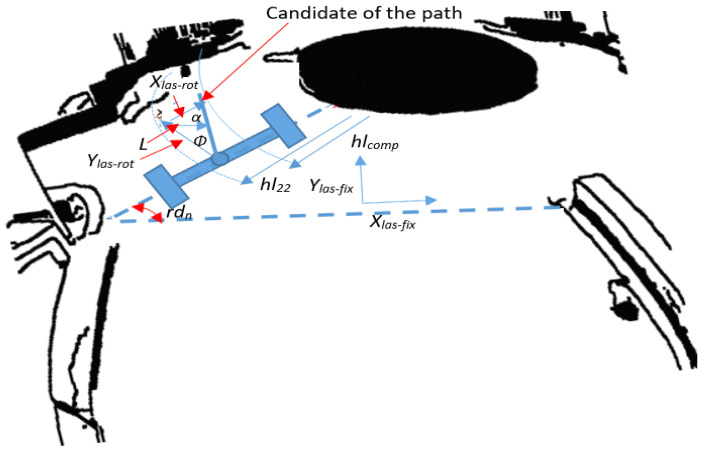
Roundabout center parameters and path determination.

In the roundabout center as shown in Figure 14, a combination between the LRF and encoders were used for rotating the robot in the correct path. Because the robot will move in a circular path, two coordinate systems can be used for describing the robot path; one is moving with the robot while the other is fixed as shown in Figure 14. The rotation will be clockwise and thus, the transformation matrix may be presented as:

(26) $$\left[\begin{array}{l} X_{las-fix}\\ Y_{las-fix} \end{array}\right]=\left[\begin{array}{l} \cos(rd-rd_{0})\;\sin(rd-rd_{0})\\ \sin(rd-rd_{0})\;\cos(rd-rd_{0)} \end{array}\right]\times \left[\begin{array}{l} X_{las-rot}\\ Y_{las-rot} \end{array}\right]$$

where *X_las-fix_*, *Y_las-fix_* is the fixed coordinate system and *X_las-rot_*, *Y_las-rot_* is the robot coordinate system. *rd* − *rd*_0_ is the total angle that robot should rotate in the roundabout.

The first measurement of LRF at the right side of robot after passing the entrance area (*hl_comp_*) was used as a reference for the robot when rotating around the roundabout, and the other LRF measurements were continuously compared to the reference to determine the (*x_las-rot_*, *y_las-rot_*) position of the robot as expressed in Equation (27):

(27) $$X_{las-rot}=hl_{22}-hl_{comp}\;Y_{las-rot}=\Delta T.\;V\;\mathrm{where}\;V=\frac{V_{r}+V_{L}}{2}$$

where *hl*22 is the current measurement of the LRF at the right side of the robot. The rotation angle *φ* can be calculated as follows:

(28) $$\varphi =\arctan\;(\frac{X_{las-rot}}{Y_{las-rot}})$$

The rotation distance of robot *L* can be calculated as:

(29) $$L=X_{las-rot}\frac{\sin(\alpha )}{\sin(\frac{\pi +\varphi }{2})}$$

where:

$$\alpha =\frac{\pi }{2}-\varphi$$

The angular velocity can be calculated as follows:

(30) $$V_{l}-V_{r}=\overset{\cdot }{\varphi }b=b\frac{\varphi_{\max}-\varphi_{0}}{t_{\max}-t_{0}}=\frac{b\varphi_{\max}}{T}=\frac{L}{T}=\frac{X_{laser}\frac{\sin(\alpha )}{\sin(\frac{\pi +\varphi }{2})}}{T}$$

**Figure 15:**
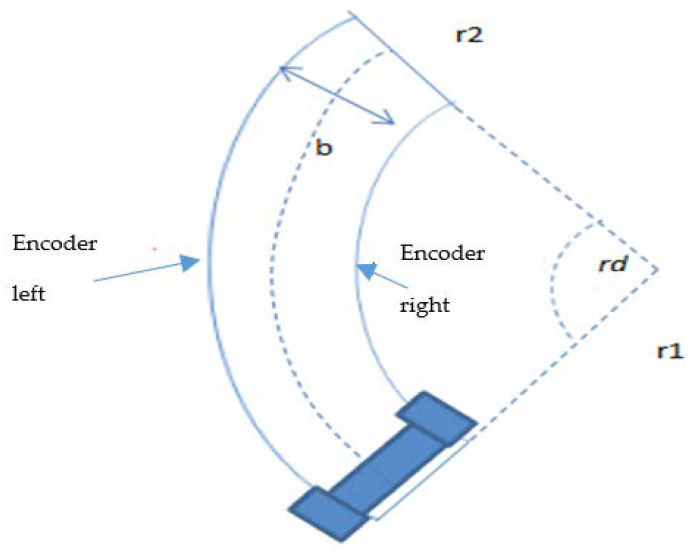
Robot rotation about the roundabout center to find the exit.

The exit of the roundabout center, where the robot stops to rotate around the roundabout, was calculated based on the encoder’s measurements. Assume that the robot moves in a circular path as shown in Figure 15. The outer and inner circumferences of this roundabout that was likened to a disk were calculated based on the positions of the right and left side’s encoders of the robot that constitutes the width (b) of the WMR.

The following equations were used to calculate the angle of rotation *rd* in rad as presented in Equation (31):

(31) $$r_{2}=r_{1}+b\;encod\_right=rd\;r_{1}\;encod\_left=rdr_{2}\;\frac{encod\_left\;}{rd}=\frac{encod\_right\;}{rd}+b\;rd=\frac{encod\_left\;-encod\_right\;}{b}$$

Four *rd* conditions can be considered:

1. *rd* > π/2: robot will exit the roundabout at the first left turn.
2. *rd* > π: robot will exit the roundabout at the second left turn and in the straight direction.
3. *rd* > 3π/2: robot will exit the roundabout at the third left turn.
4. *rd* > 2π: robot will exit the roundabout at the fourth left turn.

## 5. Results and Discussion

**Figure 16:**
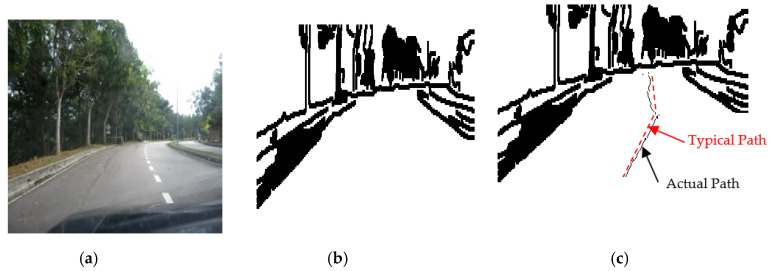
Autonomous detection and navigation of the proposed system in the road following with 5 m as width and 500 m as length: (**a**) original image, (**b**) image processing, and (**c**) generation of the path within the road following environment.

**Figure 17:**
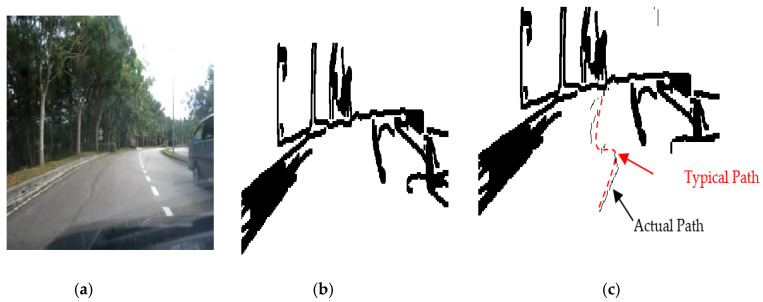
Autonomous detection and navigation of the proposed system in the road following (with 5 m as width and 500 m as length) with partial car on the side: (**a**) original image, (**b**) image processing, and (**c**) generation of the path within the road following environment.

**Figure 18:**
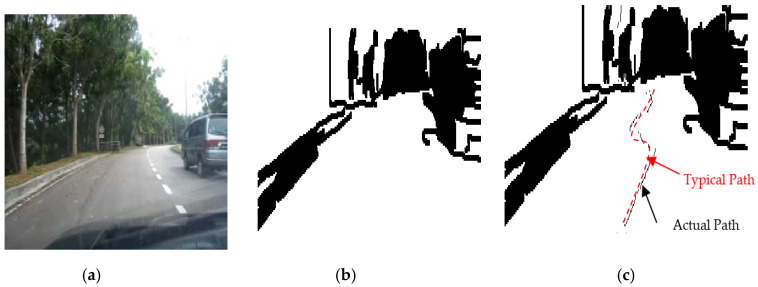
Autonomous detection and navigation of the proposed system in the road following (with 5 m as width and 500 m as length) a car partially presented on the side/in front: (**a**) original image, (**b**) image processing, and (**c**) generation of the path within the road following environment.

**Figure 19:**
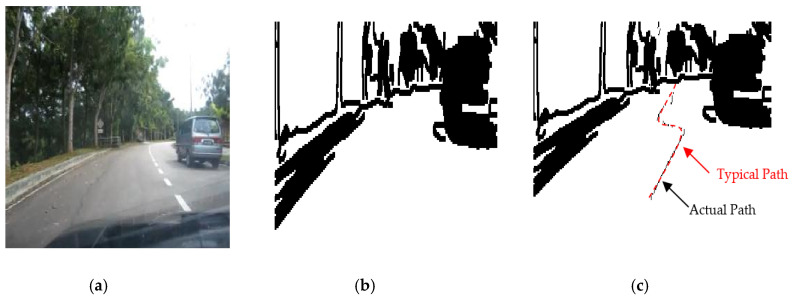
Autonomous detection and navigation of the proposed system in the road following (with 5 m as width and 500 m as length) with a car on the side/in front: (**a**) original image, (**b**) image processing, and (**c**) generation of the path within the road following environment.

**Figure 20:**
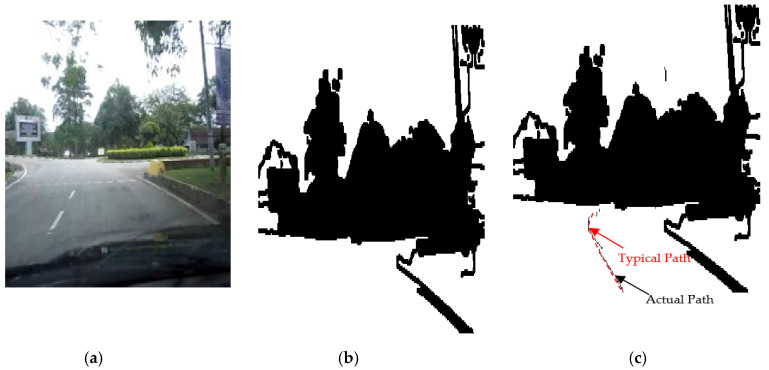
Autonomous detection and navigation of the proposed system in the road roundabout (with 5 m as diameter): (**a**) original image, (**b**) image processing, and (**c**) generation of the path within the road roundabout environment.

**Figure 21:**
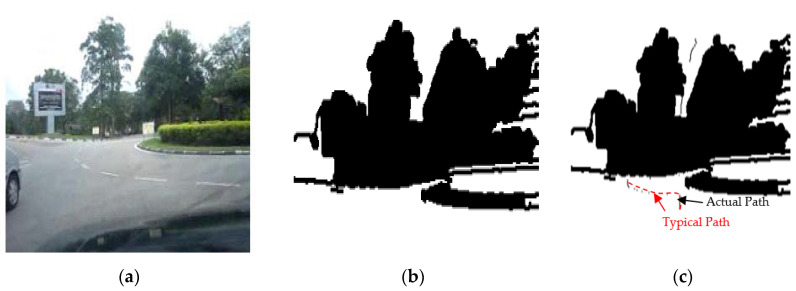
Autonomous detection and navigation of the proposed system in the road roundabout (with 5 m as diameter) with a car partially presented on the side/in front: (**a**) original image, (**b**) image processing, and (**c**) generation of the path within the road roundabout environment.

**Figure 22:**
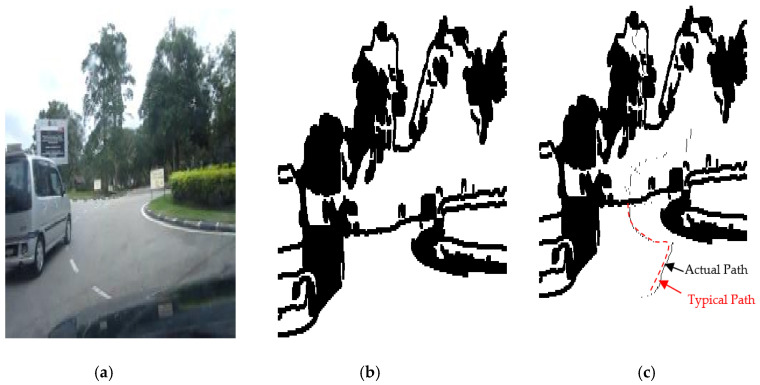
Autonomous detection and navigation of the proposed system in the road roundabout (with 5 m as diameter) with a car on the side/in front: (**a**) original image, (**b**) image processing, and (**c**) generation of the path within the road following environment.

**Figure 23:**
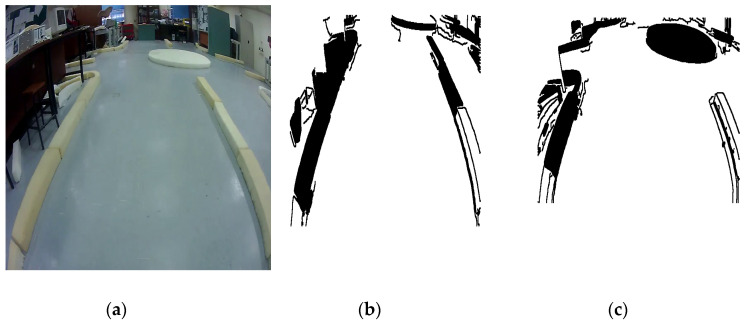
Camera sequence images: (**a**) original image when the WMR starts moving, (**b**) camera’s local map when the WMR starts to move, and (**c**) camera’s local map when the WMR detects the roundabout.

**Figure 24:**
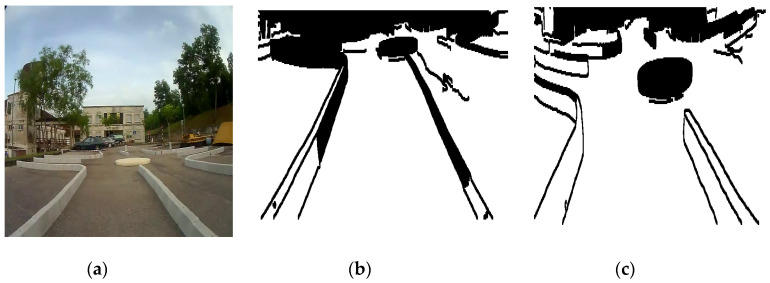
Outdoor camera sequences images: (**a**) original image when the WMR starts moving, (**b**) camera’s local map when the WMR starts to move, and (**c**) camera’s local map when the WMR detects the roundabout.

**Figure 25:**
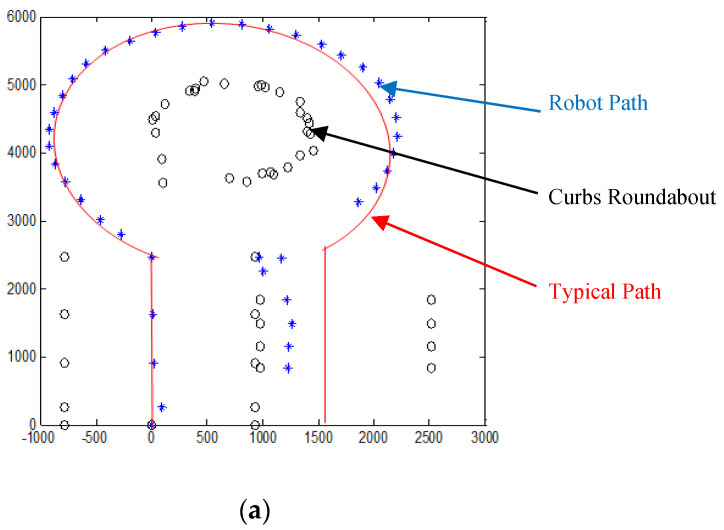

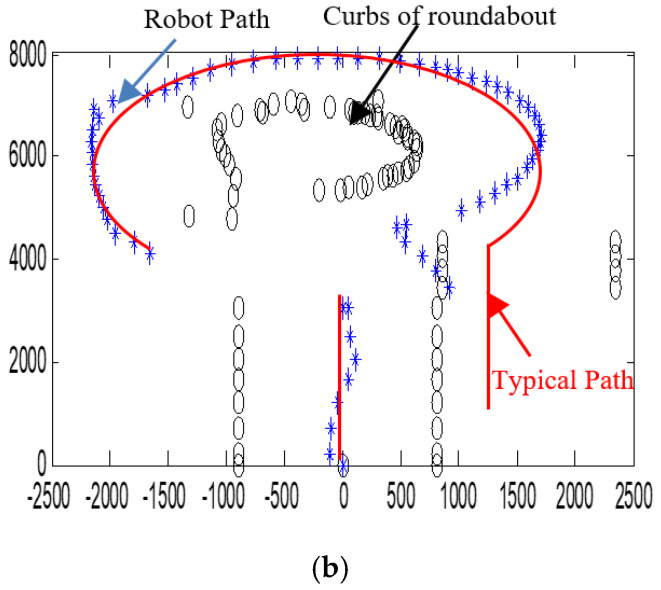
Robot path during navigation in a roundabout with 360° rotation. Note that blue ‘*’ denotes the path, and black ‘O’ signifies the road environment. (**a**) Local mapping of the indoor environment acquired by sensors fusion. (**b**) Local mapping of the outdoor environment acquired by sensors fusion.

**Figure 26:**
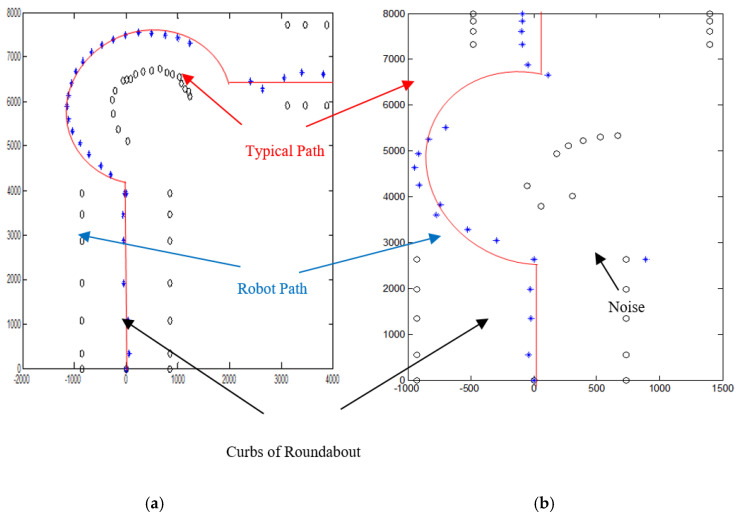

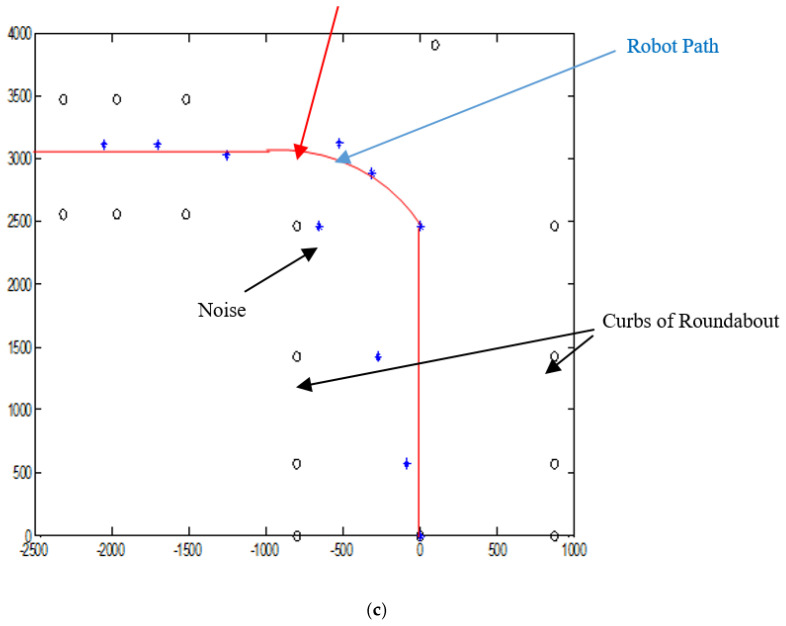
Robot path during navigation in a roundabout with 270° rotation. Note that blue ‘*’ denotes the path, and black ‘O’ signifies the road environment for: (**a**) 270° rotation, (**b**) 180° rotation, and (**c**) 90° rotation.

Several robot’s path scenarios have been performed in real road environments in both road following and roundabout cases as depicted in Figure 16, Figure 17, Figure 18, Figure 19, Figure 20, Figure 21 and Figure 22, to show the capability of the suggested algorithms for road roundabout detection and navigation. Another set of experiments have been implemented in indoor and outdoor environments to test the performance of the suggested algorithm in terms of the lighting and weather conditions changings as shown in Figure 23, Figure 24, Figure 25 and Figure 26.

In the discussion part, the results of the experiments that have been conducted in the real roads, indoor and outdoor setups are discussed based on the following aspects:

1. Accuracy of navigation system: It can be defined as the variation between the actual and typical paths during navigation in the road from start to goal position. For this purpose, the generated path (black dotted line as in Figure 16, Figure 17, Figure 18, Figure 19, Figure 20, Figure 21 and Figure 22) is compared with the typical path (red dotted line as in Figure 16, Figure 17, Figure 18, Figure 19, Figure 20, Figure 21 and Figure 22). The typical path in this work is considered as the path located in the middle of the road.
2. Efficiency (Reliability) of navigation system: It can be defined as the capability of the proposed algorithm to detect the road boundaries and borders among other surrounding environments of robot during autonomous navigation on the roads, in the presence of noise.
3. Cost of navigation system: One can differentiate between two kinds of costs, namely; fixed and operational costs. The fixed cost is the total cost of the hardware that has been used to perform the suggested algorithm, which is too low, in comparison with Tesla and Google autonomous vehicles. The total cost of robotic system in this project as shown in Figure 10 is around 5K USD; however, the cost of current autonomous vehicles such as Tesla or Google are in the range of 50–500K USD. The operational costs are varied during autonomous navigation on the roads based on the road conditions where the fuel consumption and electrical current profiles are changed during road navigation.

### 5.1. Road Following

The above-mentioned equations in Section 3.2 and Section 4.2 were applied to detect the road roundabout and find the path of mobile robots in the real road following. The path was planned to be in the middle of the LRF measurements and the robot is able to track the middle of the roads as shown in Figure 16, Figure 17, Figure 18 and Figure 19. Because of the accuracy of the LRF equal to 1 cm and the resolution (1 scan/100 ms), the robot path has a small deviation in the path as shown in Figure 16, Figure 17, Figure 18 and Figure 19.

Several scenarios for autonomous navigation of the proposed algorithms in road following (with 5 m as width and 500 m as length) has been reported:

1. The autonomous vehicle is moving lonely on the road
2. The autonomous vehicle navigation system recognizes partially other vehicles on the side/in front of autonomous vehicle, as shown in Figure 17.
3. The autonomous vehicle navigation system recognizes complete vehicles on the side/in front of the autonomous vehicle, as shown in Figure 18 and Figure 19.

The accuracy of the suggested autonomous robotic system is high, in the range of 1–3 cm, when it drives lonely on the road, as shown in Figure 16; however, it is in the range of 2–5 cm if there are obstacles on the sides or the edges cannot be detected, as shown in Figure 17, Figure 18 and Figure 19, which can be increased to 3–10 cm if there are problems in the camera and LRF, such as deblurring, processing delay, or losses in LRF signals. The efficiency of the autonomous vehicle is good as it is able to detect well the path along the movement, no matter whether there are obstacles or not; with noting that the distance between the generated dotted-path is not constant due to losses of sensors measurements and long processing time. The maximum distance between two generated dotted-path is small, around 15 cm in real road, which does not present a bad impact to the robot’s efficiency. Thus, the efficiency is in the range of 90–95% as shown in Figure 16, Figure 17, Figure 18 and Figure 19. The operational cost increases when there are obstacles beside or in front of the autonomous vehicle as the path becomes a zigzag in this case; however, it is ok when the autonomous vehicle is moving alone.

Based on Figure 16, Figure 17, Figure 18 and Figure 19, the average accuracy, efficiency, and operational cost of autonomous vehicles in road following are listed in Table 1.

### 5.2. Roundabout Intersection

The above-mentioned equations in Section 3.2 and Section 4.2 were applied to detect the road roundabout and find the path of mobile robots in the real road following. The path was planned to be in the middle of the LRF measurements and the robot is able to track the middle of the roads as shown in Figure 10. Because the accuracy and resolution of the LRF is equal to 1 cm and 1 scan/100 ms, respectively, the robot path has a small deviation in the path as shown in Figure 20, Figure 21 and Figure 22.

Similar to road following scenarios, the accuracy of the autonomous system when it is approaching the roundabout (5 m as diameter) is high, in the range of 2–3 cm, especially when it drives alone on the road as shown in Figure 20; however, it is in the range of 2–4 cm if there are obstacles on the sides, and the roundabout starts to be recognized or the edges cannot be detected as shown in Figure 21 and Figure 22. The accuracy is located in the range of 3–8 cm if there are problems in the camera and LRF, such as deblurring, processing delay, or losses in LRF signals. The efficiency of the autonomous vehicle is also good, and it is in the range of 90–95%, as shown in Figure 20, Figure 21 and Figure 22. The operational cost increases when the robot starts to recognize the roundabout or the obstacles are presented on side/in front of the autonomous vehicle.

According to Figure 20, Figure 21 and Figure 22, the average of the accuracy, efficiency, and operational cost for the autonomous vehicle when it is passing through a roundabout are listed in Table 2.

To test the reliability of detection and navigation of the roundabout algorithm in the indoor and outdoor applications, another set of experiments have been conducted as shown in Figure 23, Figure 24, Figure 25 and Figure 26.

Figure 23, Figure 24 and Figure 25 show the sensors fusion based autonomous roundabout navigation from start to goal position with rotation angle equal to 360°. Figure 23 and Figure 24 show the local map built from the camera sequence frames, where only the borders and intersections of the road remained in the images. The camera’s local map was determined for each image in the sequences of the video frames as shown in Figure 23b,c and Figure 24b,c using the LS integrated with FL algorithm, which were applied to recognize the presence of roundabouts. Figure 23c and Figure 24c show the last image in which the roundabout is detected.

By applying the algorithms described in Section 3.2 and Section 4.2, the robot path for both indoor/outdoor roundabout environments can be determined as shown in Figure 25, where the series of blue points (*) is the path and a series of black points (O) denotes the road environment, where the entrance and exit curbs of the roundabout are located at the bottom-left and bottom-right of the figure, respectively. The roundabout center is located somewhere in the middle. The robot path looks smoother in the indoor environment as shown in Figure 25a; however, there are a cleared drifts in the outdoor one as shown in Figure 25b.

Similarly, the algorithms described in Section 3.2 and Section 4.2 were applied to find the robot path in a roundabout with 270°, 180°, and 90° rotation as shown in Figure 26.

Because the Laser Range Finder is moving together with WMR platform and was simultaneously utilized to recognize (with measurements) the static road’s curbs, it was observed that there is a slight shift on the right side at the exit as depicted in Figure 25 and Figure 26. This is because the WMR platform exits from the roundabout outlet in an asymmetric manner just like when it first enters the inlet of roundabout. In addition, this drift could be related to the low accuracy of the LRF, which is about 3% of the measured distance; thus, if the measured distance is bigger than 1000 mm, the error should be in the region of 30 mm. The last reason is that the velocity of WMR is not completely controlled in these trials. In general, the main concern of this work is to present the robot trajectory rather than to show accurately the road intersections and curbs within the environment. It has been noticed that there is a noisy signal in sensor fusion measurements when they have been used to measure the distance between WMR and road curbs as depicted in Figure 25 and Figure 26. In fact, those noises are not chosen as the robot trajectory since they are located far from the truth path as shown in Figure 20b. The typical path of the root is highlighted as red color in Figure 25 and Figure 26.

### 5.3. Comparison with Other Related Works

**Figure 27:**
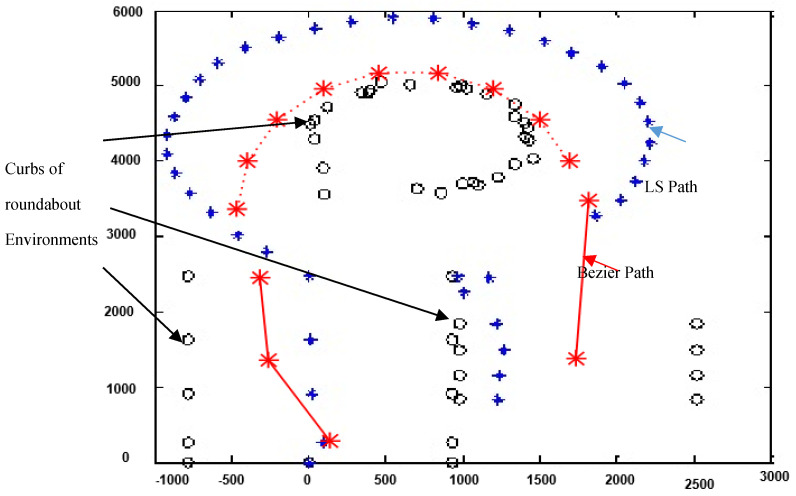
A comparison between Bezier roundabout navigation approach presented in Perez et al. [25] (red *) and the proposed roundabout navigation algorithm in this paper (blue *).

The proposed Laser Simulator–Fuzzy Logic algorithm was compared with the work presented in Perez et al. [25] to navigate WMR on the roundabout environment. As has been discussed in Section 1, the roundabout navigation algorithm in Perez et al. [25] depends mainly on the maps and GPS signals to identify the road roundabout and find its dimension, which is almost similar to Tesla’s and Google’s cars navigation system on roundabouts [30]. Once the vehicle arrives at the entrance of the roundabout, the algorithm will generate a Bezier curve to navigate off-line the vehicle in the roundabout from the inlet until outlet of roundabout. The main disadvantage of the Bezier navigation approach is its dependencies on the off-line measurements that are coming from GPS and maps to find the dimensions of the roundabout setting, which may cause the vehicle to crash with the border of the roundabout, a scenario as shown in Figure 27. Other problems of such navigation system are coming from nonupdating of the maps’ data, losing of GPS measurements in some areas, and nonregistered road roundabouts in the Google maps that could occur by making the offline path navigation in roundabout setting potentially dangerous.

The proposed Laser Simulator–Fuzzy Logic approach in this paper could resolve such issues by utilizing an online roundabout navigation scheme. The comparison between Bezier roundabout navigation approach that has been presented in Perez et al. [25] and the proposed roundabout navigation algorithm in this paper, is depicted in Figure 27, where the Bezier algorithm crashes with roundabout border at the entrance of the roundabout.

## 6. Conclusions

The online path planning of the mobile robot has been fully derived in the road following and roundabout environments using LS integrated with an FL algorithm and sensor fusion technique. The proposed algorithm was used for roundabout detection through a camera’s local map environments; while the sensor fusion was used for simultaneous planning of the robot path and building an accurate local map. The roundabout intersection was modeled, and the free-path collision was generated within its environment from the starting position located at a specific entrance to an appropriate exit of the roundabout. Results show the capability of the robot to effectively navigate in the road following and roundabout settings with multiple scenarios. Future work is to apply the signal stochastic and probabilistic methods like Kalman filter to eliminate the noise and improve the robot path. In addition, a low level control should also be applied to improve robot tracking.

## Figures and Tables

**Table 1:** An average accuracy, efficiency, and operational cost for road following with 5 m as width and 500 m as length.

Condition/ Property	Clear Road Curbs	Presence of Obstacle	Camera and LRF Problems	Missed Road Curbs
**Accuracy**	1–3 cm	2–5 cm	3–10 cm	2–5 cm
**Efficiency**	95%	90%	90%	90%
**Operational Cost**	decreased	increased	increased	Increased

**Table 2:** An average of the accuracy, efficiency, and operational cost of the autonomous vehicle when it is passing through a roundabout (with 5 m as diameter).

Condition/Property	Clear Road Curbs	Approaching to Roundabout	Presence of Obstacle	Missed Road Curbs	Camera and LRF Problem
**Accuracy**	2–3 cm	2–4 cm	2–4 cm	2–4 cm	3–8 cm
**Efficiency**	95%	90%	90%	90%	90%
**Operational Cost**	decreased	increased	increased	Increased	increased
